# Integrating Artificial Intelligence Into Drug Discovery From Medicinal Plants: Current Applications and Infrastructural Challenges

**DOI:** 10.1111/cbdd.70372

**Published:** 2026-07-31

**Authors:** Amit Gangwal, Azim Ansari, Mohd Usman Mohd Siddique, Jyotiram Sawale, Suhas Padmane, Raju Wadekar

**Affiliations:** ^1^ Department of Pharmacognosy SVKM NMIMS Global University School of Pharmacy and Technology Management Dhule Maharashtra India; ^2^ Department of Pharmaceutical Chemistry SVKM NMIMS Global University School of Pharmacy and Technology Management Dhule Maharashtra India; ^3^ Department of Pharmacognosy, Krishna Institute of Pharmacy Krishna Vishwa Vidyapeeth (Deemed to be University) Karad Maharashtra India; ^4^ Department of Pharmaceutical Quality Assurance Gurunanak College of Pharmacy Nagpur India

**Keywords:** ADMET, artificial intelligence, dereplication, drug discovery, drug repurposing, generative AI, machine learning, medicinal plants, network pharmacology, virtual screening

## Abstract

Medicinal plants represent a vast and evolutionarily refined reservoir of structurally diverse bioactive compounds that have historically contributed to major therapeutic breakthroughs. However, despite their pharmacological richness, systematic translation of plant‐derived metabolites into clinically approved drugs remains constrained by persistent bottlenecks, including extract complexity, dereplication redundancy, structural elucidation challenges, taxonomic ambiguity, and multi‐component pharmacology. This review presents a bottleneck‐driven and systems‐oriented framework for integrating artificial intelligence (AI) into medicinal plant‐based drug discovery (MPDD) to address some of these bottlenecks. Rather than reiterating broadly documented AI tools used in synthetic drug development, the manuscript critically examines phytomedicine‐specific challenges and maps AI applications across key stages of the pipeline: medicinal plant identification, extraction optimization, plant metabolite identification and dereplication, absorption, distribution, metabolism, excretion, and toxicity (ADMET) prediction, virtual screening, network pharmacology, repurposing, and generative de novo design of pseudo‐natural products. Emphasis is placed on the foundational requirement of making phytochemical datasets truly AI‐ready through metadata harmonization, dataset balancing, novelty‐aware modeling, and structured data engineering. Emerging approaches such as transformer‐based foundational models, graph neural networks (GNNs), generative AI (GAI) architectures, and multi‐omics‐integrated network pharmacology are discussed within a pragmatic translational context. Importantly, this review maintains a balanced perspective, acknowledging that fully AI‐driven clinically approved botanical drugs/synthetic drugs have yet to emerge and identifies challenges limiting the progress. By integrating computational methods with infrastructural reform, this work outlines a progressive roadmap to transition AI in medicinal plant research from exploratory studies toward standardized, reproducible, and biologically grounded next‐generation drug discovery.

## Graphical abstract

This review presents a systems‐oriented roadmap for integrating artificial intelligence into medicinal plant drug discovery to overcome persistent bottlenecks like extract complexity. It highlights that advancing toward reproducible therapeutics requires making phytochemical datasets AI‐ready via rigorous harmonization and phyto‐centric foundational models.

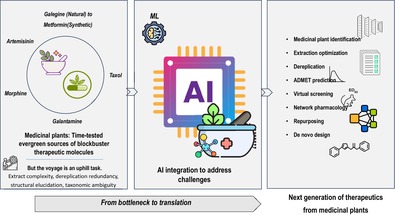

**Abbreviations**
ACBA: acetylbinankadsurin A
AChE: acetylcholinesterase
ADMET: absorption, distribution, metabolism, excretion, and toxicity
AHC: augmented hill‐climb
AI: artificial intelligence
ANN: artificial neural networks
APIs: active pharmaceutical ingredients
BGCs: biosynthetic gene clusters
CNNs: convolutional neural networks
DFT: density functional theory
DL: deep learning
DNN: deep neural network
DOGS: design of genuine structures
GAI: generative AI
GNNs: graph neural networks
HCC: hepatocellular carcinoma
H‐C‐T‐D: herb‐compound‐target‐disease
HTS: high‐throughput screening
MAE: microwave‐assisted extraction
MAFLD: metabolic dysfunction‐associated fatty liver disease
MD: molecular dynamics
ML: machine learning
MPDD: medicinal plant‐based drug discovery
MS: mass spectrometry
MS/MS: tandem mass spectrometry
NIMO: natural product‐inspired molecular generative model
NLP: natural language processing
NMR: nuclear magnetic resonance
NUS: non‐uniform sampling
OED: orthogonal experimental design
OSCC: oral squamous cell carcinoma
PAINS: pan‐assay interference compounds
PCI: Pharmacy Council of India
QGJZF: Qigui Jiangzhi Formula
QSAR: quantitative structure–activity relationship
RF: random forest
RNN: recurrent neural networks
RXR: retinoid X receptor
SVM: support vector machine
UASE: ultrasound‐assisted solvent extraction
VAEs: variational autoencoders
VC: vascular calcification
XO: xanthine oxidase

## 1 Introduction

**FIGURE 1:**
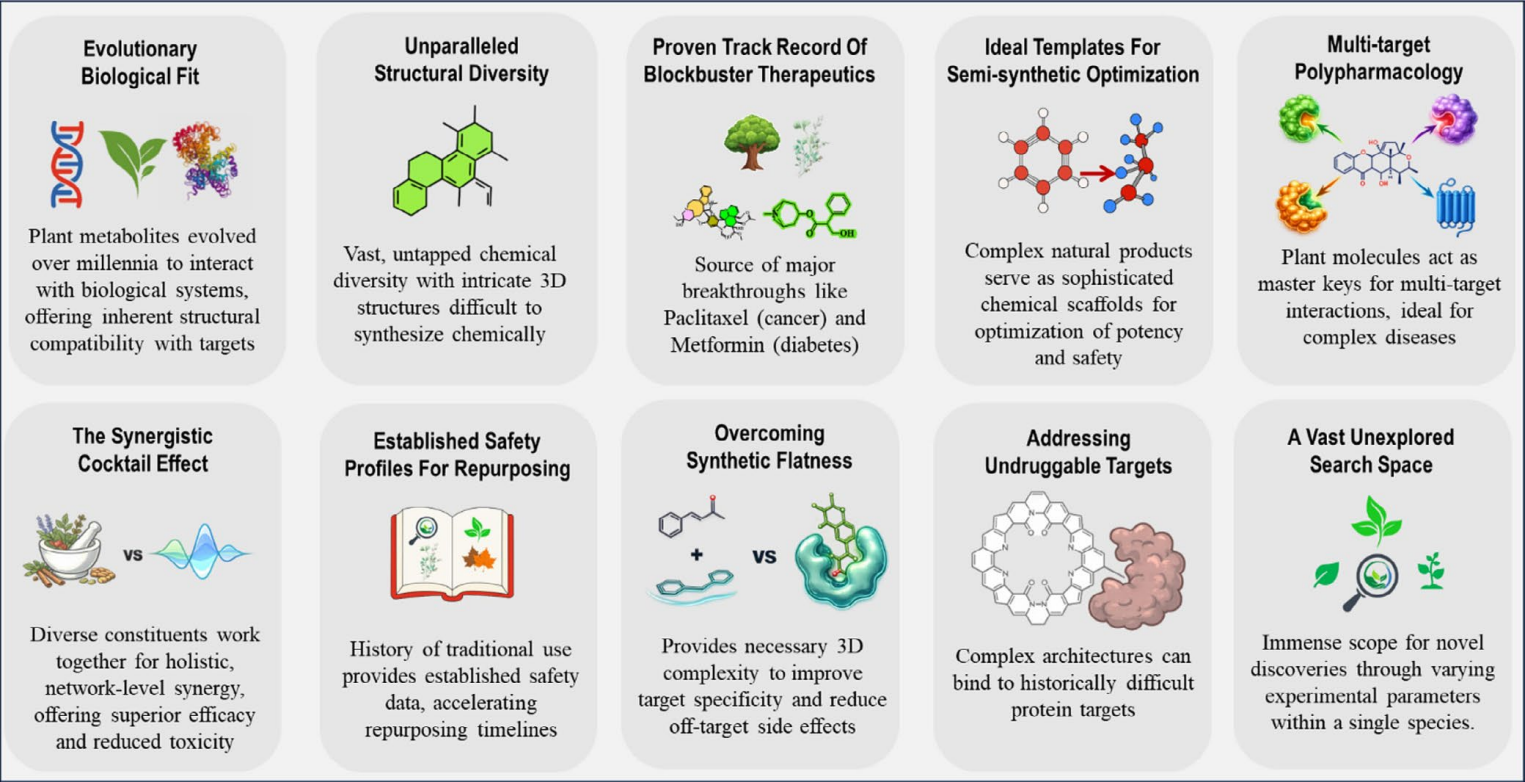
A self‐explanatory visual summary of most compelling reasons why medicinal plants remain the premier source for new therapeutics.

For decades, the field of drug discovery has been predominantly driven by synthetic chemistry, a highly successful paradigm that has delivered the vast majority of modern medicines through precise, rational design. However, the plant kingdom represents a complementary and largely untapped reservoir of chemical diversity (Newman and Cragg 2007). Unlike synthetic libraries, which are often designed around known chemical space, medicinal plants offer structurally complex secondary metabolites that have evolved over millennia to interact with biological systems. These natural products serve as ready‐to‐prescribe drugs and invaluable chemical scaffolds. They provide structural templates for semi‐synthetic optimization and create opportunities for drug repurposing, giving established molecules new therapeutic uses. Ultimately, this approach maximizes nature's library while significantly reducing drug development timelines (Atanasov et al. 2015). Additionally, terrestrial medicinal plants are easier to explore for drug discovery than marine organisms or microbes. This is mainly due to their easy accessibility, simple cultivation, and extensive ethnobotanical knowledge. Marine environments often require specialized and expensive equipment for exploration. In contrast, terrestrial plants can be readily located and collected. Many medicinal plants can also be cultivated on a large scale for research and production. Microbial sources present different challenges. Many wild microbes cannot be easily grown under standard laboratory conditions. Moreover, their drug‐producing genes often remain inactive outside their natural environments. Another important advantage of terrestrial plants is their long history of use in traditional medicine. Thousands of years of human experience provide valuable clues about their biological activities. This knowledge helps researchers prioritize promising species for investigation. As a result, the screening process becomes more efficient. Such guidance is generally unavailable when exploring deep‐sea organisms or microscopic ecosystems (Figure 1).

The profound impact of this natural inspiration is evident from various pharmacopoeias. The classic cancer drug paclitaxel (Taxol), rooted in the bark of *Taxus brevifolia* , revolutionized oncology by stabilizing microtubules, a mechanism previously unknown to science (Schiff et al. 1979). Additionally, the molecule was novel and not a derivative or altered version of some known molecule/scaffold. Similarly, the global standard for diabetes, metformin, traces its lineage to the plant *Galega officinalis* , proving that medicinal plants are often the essential starting points for entire classes of synthetic derivatives (Bailey 2017). Yet, despite these successes, the systematic translation of medicinal plant‐derived compounds into clinical drugs remains challenging.

**FIGURE 2:**
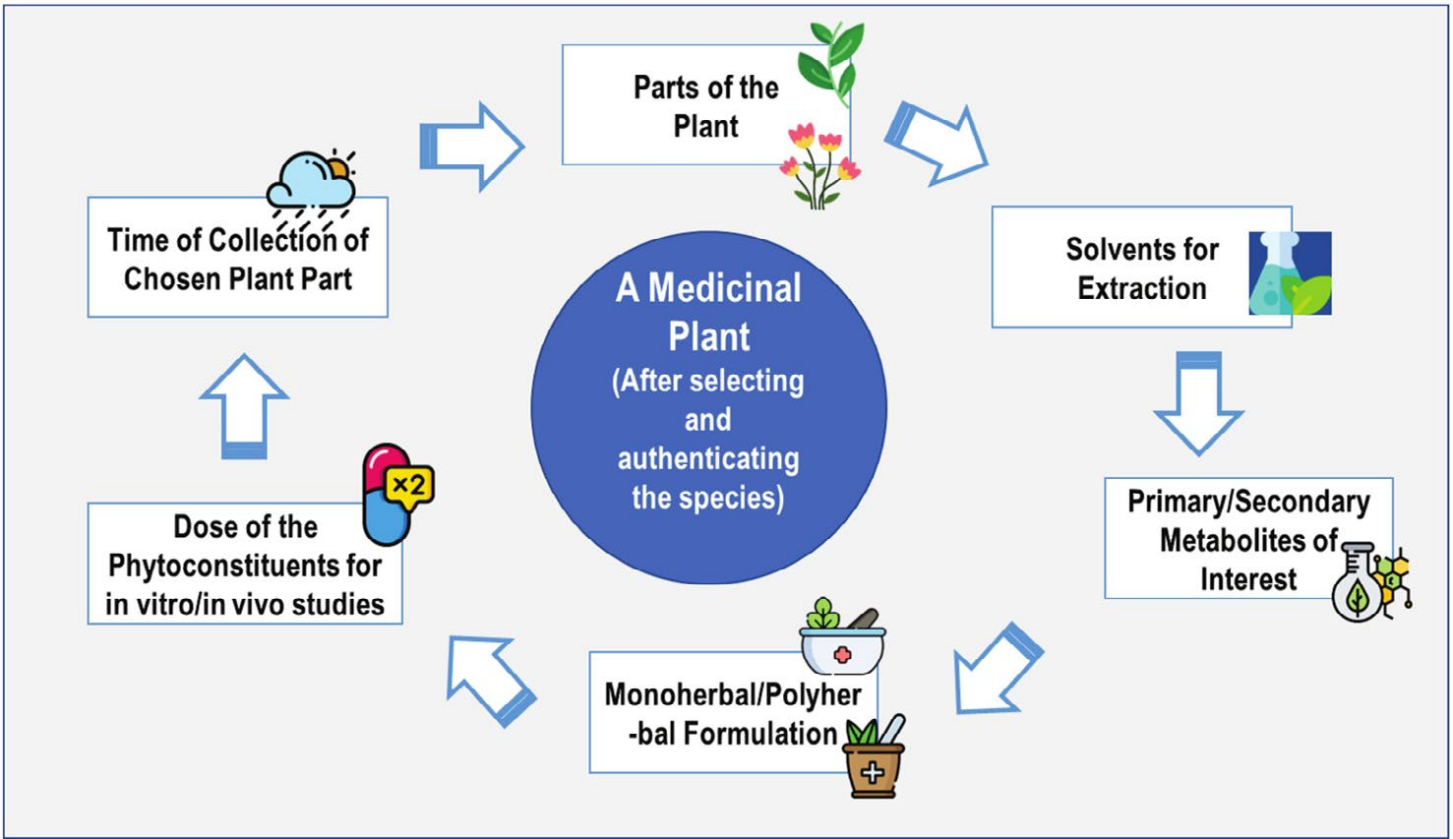
Exploring the multidimensional search space of a single medicinal plant to uncover novel phytochemicals through methodological variations.

The field is constrained by inherent bottlenecks: the high chemical complexity of crude extracts, the labor‐intensive nature of isolation, and the persistent issue of dereplication (the struggle to rapidly identify known compounds to avoid redundant efforts). Furthermore, unlike static synthetic leads, plant chemistry is a dynamic matrix, varying significantly with environmental conditions and processing methods (Koehn and Carter 2005). Figure 2 illustrates why rapid screening of plants (jumping from species to species) often misses therapeutic leads and why the exhaustive exploration of a single species offers a vast, often underestimated scope for novel phytochemicals.

In response to these challenges, AI (machine learning‐ML and deep learning‐DL) are emerging as supportive tools (Ydyrys et al. 2025). In the prioritization phase, AI assists in the data‐driven selection of medicinal plants, moving beyond serendipity to evidence‐based screening. During the benchwork phase, it aids in extraction and process optimization, while advanced algorithms drive metabolite annotation and dereplication, helping scientists untangle complex mixtures quickly (Wang et al. 2016). Beyond isolation, AI is reshaping mechanism elucidation through network pharmacology‐based analysis, which maps the multi‐component interactions typical of medicinal plant‐based formulations (Cui et al. 2025; Zhang et al. 2020). AI is also accelerating virtual screening and ADMET prediction, allowing for early assessment of safety and efficacy. The rise of GAI is enabling novel hypothesis generation, guiding the design of synthetic derivatives. Repurposing is another emerging area where AI algorithms are being tried (Gangwal, Ansari, Ahmad, Azad, Kumarasamy, et al. 2024).

However, the integration of AI into medicinal plant research is still in its infancy. Additionally, it is critical to note that AI is not a replacement for experimental workflows but rather a facilitator that enhances decision‐making at multiple stages of the pipeline. Current applications are largely exploratory and face significant hurdles, including the scarcity of curated datasets, the difficulty of digitizing traditional knowledge, and the black box nature of many models (Xu et al. 2025; Patil et al. 2024).

While recent reviews have explored the intersection of AI and natural‐product research, most focus on specific AI applications, such as virtual screening, network pharmacology, or natural‐product discovery, and often provide descriptive overviews of ML methodologies. In contrast, the present review adopts a bottleneck‐driven and systems‐oriented perspective. It focuses specifically on MPDD rather than natural products in general. The review examines the entire AI‐enabled drug discovery pipeline. It covers plant identification and extraction optimization. It also discusses phytoconstituent characterization and dereplication. In addition, the review explores AI applications in ADMET prediction and drug repurposing.

Recent advances in GAI are also highlighted. Furthermore, the role of network pharmacology in medicinal plant research is discussed. To maintain a focused narrative, we intentionally avoid extensively revisiting well‐established topics, including general AI methodologies, training databases, biosynthetic gene clusters (BGCs), and broader synthetic drug discovery workflows, which have been comprehensively reviewed elsewhere. Instead, we concentrate on the unique chemical, biological, and translational challenges associated with MPDD.

A key distinguishing feature of this review is its emphasis on the infrastructural and data‐related requirements necessary to make phytomedicine research genuinely AI‐ready. In particular, we discuss how rigorous dataset engineering through metadata harmonization, dataset balancing, and novelty‐aware modeling can address persistent challenges such as annotation noise, taxonomic ambiguity, and algorithmic bias. Furthermore, we highlight emerging opportunities including multimodal learning systems, phyto‐centric foundation models, and standardized experimental workflows as potential drivers of future progress. While recognizing the transformative potential of AI, we maintain a pragmatic perspective on the current translational landscape, acknowledging the limitations imposed by incomplete biological data and the continued reliance on experimental validation. By integrating technological advances with the foundational requirements needed for their successful implementation, this review aims to provide a practical and forward‐looking roadmap toward robust, reproducible, and biologically grounded next‐generation medicinal plant drug discovery.

## 2 The Bottlenecks of MPDD

Despite the historical success of plant‐derived therapeutics, the integration of medicinal plant‐derived compounds into modern drug discovery pipelines has been severely hampered by intrinsic technical challenges (Atanasov et al. 2021). The primary impediment is the high chemical complexity of crude extracts. Plant metabolomes are exceedingly complex, often containing thousands of specialized metabolites with varying polarities, stabilities, and concentrations (Najmi et al. 2022). In many cases, the bioactive constituent is a minor trace element masked by the metabolic noise of abundant, pharmacologically inert compounds (e.g., tannins, pigments, polysaccharides, etc.). This matrix interference frequently leads to false positives in high‐throughput screening (HTS) campaigns or antagonistic interactions that obscure the efficacy of the active agent.

This complexity directly necessitates labor‐intensive isolation and characterization workflows. Traditional bioassay‐guided fractionation is a rate‐limiting step, often requiring repeated cycles of chromatographic separation to purify the active principle. The process is not only time‐consuming but also resource‐heavy, requiring significant biomass. Furthermore, the structural elucidation of these isolates (often possessing intricate stereochemistry and multiple chiral centers) demands sophisticated spectroscopic analysis, which slows the hit‐to‐lead timeline significantly compared to the rapid optimization cycles possible in synthetic combinatorial chemistry (Table 1).

**TABLE 1:** Key differences between medicinal plant–derived and synthetic drug discovery approaches.

Feature	Drug discovery from medicinal plants	Synthetic drug discovery
Starting material	Complex Mixtures: Extracts contain hundreds of unknown compounds (primary and secondary metabolites from medicinal plants). Finding the single active needle in the haystack is the primary task	Defined Libraries: High‐purity, specific chemical libraries (thousands to millions of compounds) with known structures are used
Process	Bioactivity‐guided Fractionation: One must separate the extract into smaller fractions, test each for activity, and repeat until the pure molecule is isolated	HTS: Automated robots test thousands of pure compounds against a target (protein/enzyme) rapidly to find hits
Structural complexity	High: Plant molecules often have complex 3D structures (stereochemistry/chirality) and rigid scaffolds that are difficult to synthesize artificially	Variable: Synthetic molecules are often designed to be simpler and flatter to ensure they are easy to manufacture
Primary difficulty	Dereplication & Isolation: It is difficult to avoid rediscovering known compounds. Also, active compounds are often present in minute quantities, requiring massive amounts of raw plant material	Target Specificity: Synthetic drugs often lack the evolutionary fit that plant molecules have, leading to issues with toxicity or lack of selectivity (off‐target effects)
Supply & reproducibility	Low Consistency: Plant composition varies by season, geography, and climate. Securing a sustainable supply of the raw plant without driving it to extinction is a major hurdle	High Consistency: Synthesis is highly reproducible and scalable in a factory setting, ensuring exact batch‐to‐batch consistency
Optimization (SAR)	Difficult: Modifying a complex natural molecule to make it a better drug is chemically challenging due to its intricate structure	Efficient: Synthetic scaffolds are designed to be easily modified, allowing chemists to quickly create hundreds of variations to optimize the drug
Time & cost	Slower & Costly: The isolation and structure elucidation process is time‐consuming (months to years) before pre‐clinical studies/clinical trials can even be considered	Faster Screening: HTS can screen millions of compounds in weeks, though moving from a hit to a clinical candidate still takes years

Compounding the isolation challenge is the persistent issue of dereplication. Without robust early‐stage identification strategies, significant resources are frequently expended isolating new actives, only for the workflow to ultimately reveal that the compound is a known entity. This redundancy not only creates a high attrition rate and diminishes the return on investment for screening programs, but also generates more unproductive literature. While advances in LC–MS/MS and molecular networking have improved the ability to annotate known compounds early, the rediscovery of known scaffolds remains a substantial efficiency drain (Wang et al. 2016). In one of the recently published papers, authors explicitly mentioned the reason behind not pursuing/exploring few of the most common flavonoids further for docking, as ample quanta of data are there (Gangwal et al. 2026).

A critical block in early‐stage botanical drug discovery is the strategic selection of candidate medicinal plants, as both ubiquitous and relatively obscure species have often been subjected to extensive, multi‐disciplinary investigations. Consequently, determining whether a specific pharmacological activity has received insufficient scientific attention proves exceedingly difficult. This challenge of literature saturation is severely compounded by the fragmentation and inconsistency of existing phytochemical databases (Search the Databases, n.d.).

For any given botanical species, disparate repositories frequently report conflicting chemical profiles, lacking consensus on both the specific identity and the total number of documented secondary metabolites. Ultimately, this lack of standardized, comprehensive metabolomic data hampers the reliable deployment of AI‐driven predictive models, complicating the systematic selection and computational evaluation of novel phytotherapeutic leads.

Finally, even when a novel bioactive lead is identified, difficulties in synthesis and scale‐up often prevent clinical progression. Many potent plant secondary metabolites possess architectures so structurally complex, replete with macrocycles or fused ring systems, that total synthesis is not commercially viable (Zalessky et al. 2024). Consequently, supply relies on extraction from the source plant, which is often constrained by low natural yields (for instance, taxol, vincristine, etc.), slow growth rates, geopolitical access issues, and seasonal variability in metabolite production. This supply problem creates a bottleneck in drug development. A promising lead cannot be produced in sufficient quantities for pre‐clinical toxicology or clinical trials. This limitation persists until a robust, reproducible, and easy wet‐lab synthesis becomes feasible. However, achieving such a synthesis is itself a major research challenge. As a result, development timelines can be significantly delayed.

Developing a new drug is a high‐stakes endeavor that historically takes over a decade and costs billions, with a 90% failure rate in clinical trials (Gangwal and Lavecchia 2024). In this context, integrating AI is no longer just an advantage; it has become a necessity to overcome these persistent bottlenecks. While it has not yet yielded an immediate torrent of approved medicines, AI provides the essential computational power needed to navigate biological complexity, optimize designs, and de‐risk the arduous path from laboratory concept to clinical reality.

## 3 AI in MPDD

The trajectory of drug discovery from medicinal plants has evolved through distinct historical epochs, transitioning from empirical traditional practices to advanced computational paradigms. In primitive times, the use of flora for therapeutic purposes was guided by serendipity, trial‐and‐error, and deep‐rooted ethnobotanical wisdom, forming the empirical foundation of ancient healthcare systems like Ayurveda and Traditional Chinese Medicine. The 19th and 20th centuries marked a pivotal shift with the advent of modern pharmacognosy and reductionist science, characterized by the physical isolation of single active pharmaceutical ingredients (APIs) from crude extracts. This era yielded monumental therapeutic breakthroughs, as mentioned in Introduction. However, as the pharmaceutical industry increasingly favored synthetic combinatorial chemistry and HTS in the late 20th century, botanical drug discovery experienced a decline. This stagnation was primarily driven by reasons mentioned in Section 2.

Today, the field is undergoing a digital renaissance. To overcome these historical wet‐lab limitations, AI is emerging as a supportive framework for medicinal plant research by bridging ancient ethnobotanical wisdom with data‐driven, systems‐level drug discovery to accelerate the entire pipeline from plant selection to lead optimization (Saripalli et al. 2025). For instance, by integrating high‐throughput omics data, molecular networks, and biological pathways, AI assists in modeling the complex multi‐component, multi‐target interactions of herbal medicines (Suryawanshi et al. 2026) Similarly, AI‐powered dereplication uses DL models to analyze LC–MS and NMR spectroscopic data. This helps to rapidly identify known compounds. It also prevents redundant discovery. In addition, ML‐based quantitative structure–activity relationship (QSAR) models and molecular docking are used. These approaches predict bioactivity and ADMET (pharmacokinetic) properties (Johnson and Tipirneni‐Sajja 2024; Nazarenko et al. 2016). GAI broadens current capabilities by proposing natural‐product derivatives, while natural language processing (NLP)‐based mining of traditional literature and computer vision–assisted plant identification further help researchers (Othman et al. 2025).

AI optimizes green extraction processes (Mighri et al. 2026), automates quality control workflows, and plays a critical role in drug repurposing by identifying new applications for known botanical compounds.

Several successful implementations of AI algorithms corroborate these applications. For instance, to provide a cost‐effective, large‐scale screening tool for drug discovery, a partially connected deep neural network (DNN) was designed to identify medicinal uses for natural compounds. The methodology integrated latent knowledge from text mining, molecular interactions, and chemical properties to process heterogeneous data. This architecture effectively overcomes the bottleneck of incomplete information typical of plant metabolites, significantly outperforming traditional ML methods and achieving a high average AUROC of 0.9 (Yoo et al. 2020).

Another research group applied ML algorithms (support vector machine‐SVM, Random Forest‐RF, and nearest neighbor pipelines) to evaluate the immunomodulatory potential of over 200 medicinal plants. Bypassing the slow and costly nature of traditional experimental assays, the optimized models identified key active compounds with 80% accuracy. These computational predictions were subsequently validated by laboratory experiments, underscoring the efficiency of ML in accelerating the discovery of natural immunomodulators (Zerouali and Rhinane 2026).

**FIGURE 3:**
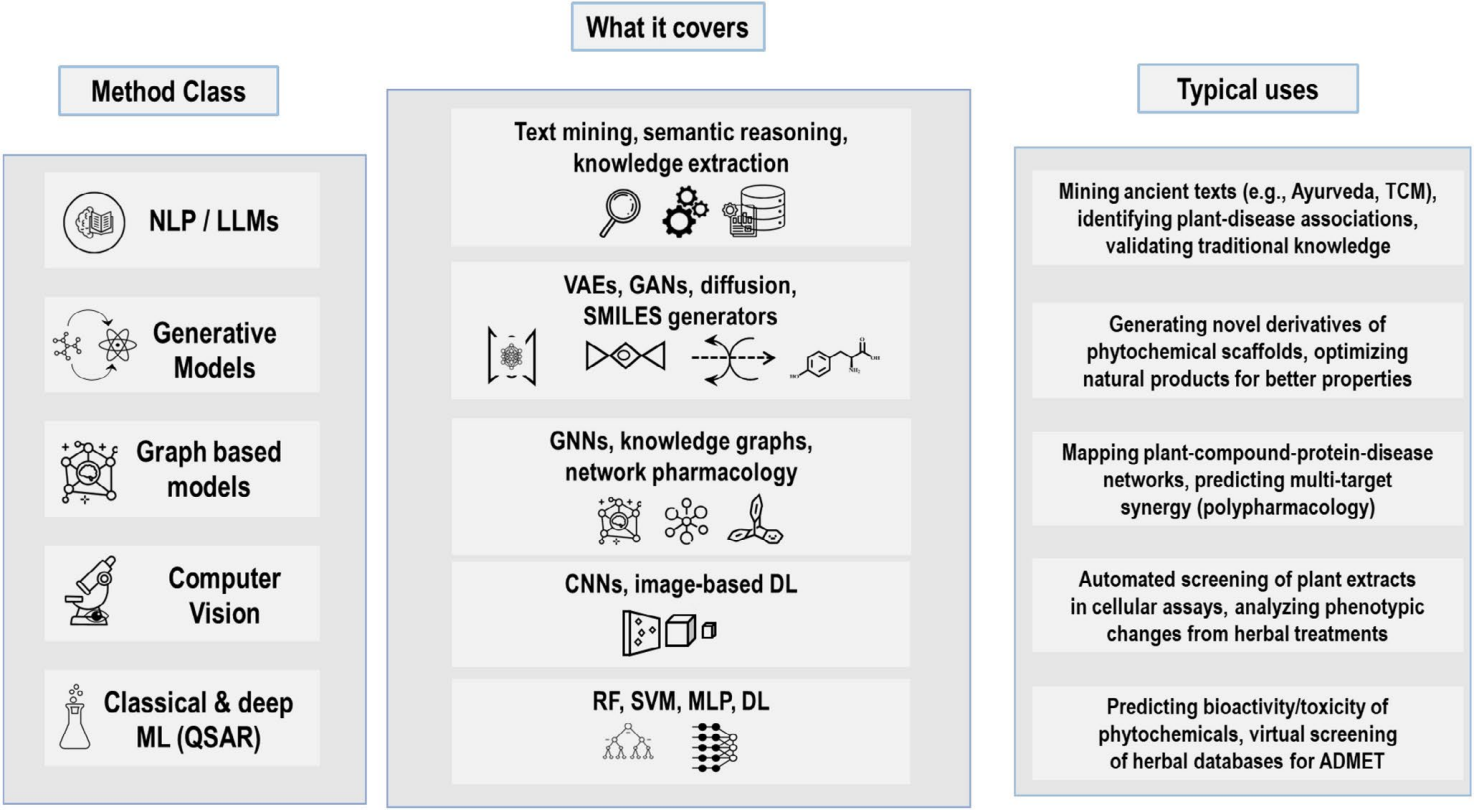
Mapping AI Methods to MPDD. This guide categorizes the primary AI architectures used in medicinal plant research. It maps specific algorithmic classes, such as NLP, Generative Models, GNN, Computer Vision, and Classical ML, to their distinct practical applications.

A bias‐corrected SVM significantly outperformed traditional ethnobotanical methods in predicting antiplasmodial properties across three plant families. The AI model identified over 7600 promising species, including 1300 active plants likely missed by conventional pharmacognosy approaches, highlighting a vast, untapped resource for novel antimalarial therapeutics (Richard‐Bollans et al. 2023). Figure 3 shows core AI techniques driving modern natural product drug discovery.

**FIGURE 4:**
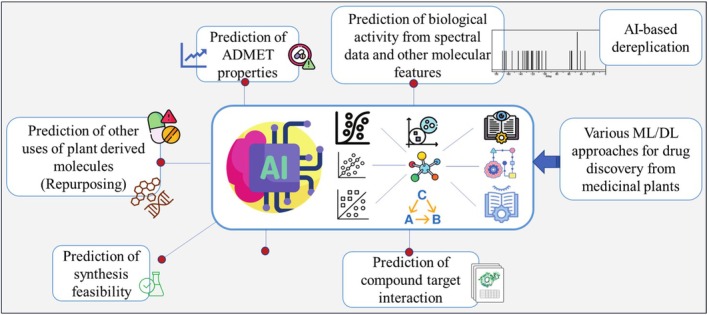
Overview of AI applications in drug discovery from medicinal plants.

In this section, various applications of AI in MPDD are being discussed with various case studies (Figure 4).

### 3.1 Selecting Appropriate AI Models for Phytochemical Datasets: A Critical Perspective

Although numerous AI algorithms have been applied in MPDD, no single model is universally optimal for all phytochemical datasets. The performance of an AI model is strongly influenced by the nature of the available data, including dataset size, feature dimensionality, molecular representation, degree of annotation, and biological complexity. Consequently, selecting an appropriate algorithm is often more important than merely increasing model complexity.

For relatively small and structured phytochemical datasets, classical ML methods such as RF, SVM, and Gradient Boosting algorithms frequently outperform DL approaches. Medicinal plant datasets often contain only hundreds or a few thousand compounds, making them vulnerable to overfitting when highly parameterized neural networks are employed. RF models are particularly effective because they can handle nonlinear relationships, tolerate noisy variables, and provide feature‐importance rankings that improve interpretability. Similarly, SVM models perform well in high‐dimensional descriptor spaces and have repeatedly demonstrated robust performance for bioactivity prediction and medicinal plant classification tasks.

DL architectures become advantageous when large‐scale datasets or high‐dimensional unstructured data are available. CNN are particularly suitable for image‐based applications such as medicinal plant identification, disease diagnosis, and microscopic analysis because they automatically learn hierarchical visual features without manual feature engineering (Gangwal and Lavecchia 2024). Transformer‐based architectures and large language models (LLMs) are increasingly valuable for mining ethnopharmacological literature, extracting plant–disease relationships, and integrating textual knowledge with molecular information.

For molecular property prediction and target identification, GNNs often provide superior performance compared with conventional descriptor‐based approaches. Unlike traditional ML models that rely on predefined molecular descriptors, GNNs operate directly on molecular graphs and preserve structural information regarding atoms, bonds, stereochemistry, and connectivity (Jiang et al. 2021). This characteristic is especially important for phytochemicals, which frequently possess highly complex scaffolds, extensive stereochemistry, and multiple ring systems that may not be adequately represented by conventional fingerprints.

Generative models including variational autoencoders (VAEs), generative adversarial networks (GANs), diffusion models, and transformer‐based molecular generators are emerging tools for de novo design of pseudo‐natural products (Bilodeau et al. 2022). However, despite their capacity to explore novel chemical space, their practical application remains limited by insufficient phytochemical training datasets, synthetic feasibility concerns, and the frequent generation of chemically implausible structures. Consequently, generative models currently serve as hypothesis‐generation tools rather than fully autonomous drug‐design platforms.

Overall, current evidence suggests that RF and SVM models remain highly effective for small‐to‐medium phytochemical datasets. CNNs dominate image‐based plant identification tasks. GNNs represent the most promising architecture for molecular prediction. They are also promising for network pharmacology. Transformer‐based models are likely to become increasingly important. They can integrate multimodal phytochemical, biological, and ethnobotanical data. Therefore, future progress in AI‐enabled medicinal plant drug discovery will depend not only on algorithmic innovation but also on the development of larger, standardized, and phytochemistry‐specific datasets capable of supporting advanced DL frameworks.

As no single AI framework is universally optimal across all stages of MPDD, understanding the strengths and limitations of individual approaches is essential for their effective implementation. Table 2 provides a comparative overview of major AI methodologies, highlighting their preferred dataset types, key applications, advantages and limitations in phytochemical research.

**TABLE 2:** Comparative assessment of AI approaches for MPDD and phytochemical datasets.

AI approach	Dataset type most suitable	Primary MPDD application	Major advantages	Major limitations
RF	Small‐to‐medium structured phytochemical datasets	Bioactivity prediction, QSAR, ADMET prediction, extraction optimization	Robust against overfitting, handles noisy data, interpretable feature importance	Performance may plateau for highly complex relationships
SVM	High‐dimensional datasets with limited sample sizes	Bioactivity prediction, medicinal plant classification, virtual screening	Strong performance in small datasets, effective nonlinear classification	Computationally expensive for very large datasets
Gradient Boosting/XGBoost	Structured descriptor‐based datasets	QSAR, activity prediction, extraction optimization	High predictive accuracy and feature selection capability	Lower interpretability compared with RF
Artificial Neural Networks (ANNs)	Medium‐to‐large structured datasets	Virtual screening, activity prediction, extraction optimization	Captures complex nonlinear relationships	Requires larger datasets and parameter tuning
DNNs	Large molecular datasets	Virtual screening and drug‐target prediction	Learns complex molecular patterns automatically	Risk of overfitting in limited phytochemical datasets
CNNs	Image datasets	Plant identification, disease diagnosis, microscopic analysis	Automated feature extraction and high classification accuracy	Sensitive to image quality and dataset bias
NLP/LLMs	Ethnobotanical and scientific literature	Ethnobotanical mining, knowledge extraction	Rapid processing of large textual datasets and hidden relationship discovery	Dependent on literature quality and database coverage
GNNs	Molecular graph datasets	Network pharmacology, target prediction, ADMET prediction	Preserves molecular topology, stereochemistry, and connectivity	High computational cost and data requirements
ML‐Assisted Optimization	Experimental process datasets	Extraction optimization (MAE, UAE, SFE)	Reduces experimental workload and resource consumption	Dependent on quality of experimental input data
AI‐Assisted Dereplication	LC–MS/MS, NMR, metabolomics datasets	Metabolite annotation and compound identification	Accelerates identification of known compounds and reduces rediscovery	Limited by spectral database completeness
GAI (GANs, VAEs, Diffusion Models, Transformers)	Large molecular datasets	De novo design of pseudo‐natural products	Generates novel chemical entities and explores new chemical space	Limited experimental validation and synthetic feasibility concerns
Integrated AI + Network Pharmacology	Multi‐omics and biological interaction datasets	Multi‐target drug discovery and mechanism elucidation	Captures synergistic and systems‐level effects of phytochemical mixtures	Requires extensive and high‐quality biological datasets

### 3.2 Plant Identification and Ethnobotanical Mining

The initial phase of MPDD, selecting the right plant and correctly identifying it, has historically been plagued by taxonomic ambiguity. AI is currently addressing these upstream bottlenecks through computer vision (Darshana and Soumyakanta 2022).

To operationalize these broad computer vision capabilities and overcome the persistent challenge of visual similarity among related species, researchers are increasingly relying on DL architectures. Consequently, DL, specifically convolutional neural networks (CNNs), have revolutionized this domain by automating morphological analysis, bypassing the scarcity of expert taxonomists. Unlike traditional chemical profiling, which is destructive and time‐consuming, CNNs can analyze leaf venation, texture, and shape in real‐time (Musyaffa et al. 2023). A pertinent example of this application is the work by Malik et al. (2022), who deployed a DL architecture to identify medicinal flora in biodiversity hotspots. This type of ML‐based model has scaled beyond academic niche use, with platforms like Pl@ntNet validating the utility of visual AI in processing millions of botanical images for species confirmation. Pl@ntNet operates as a citizen science–based plant identification system that applies ML techniques to analyze photographs submitted by users (Neto‐Bradley et al. 2026).

To address the issue of small datasets, controlled imaging conditions, and inadequate handling of real‐world variability, Firdous et al. (2026) have developed a hybrid approach combining a pre‐trained vision transformer with an Optuna‐tuned CatBoost classifier, achieving 93% training accuracy and demonstrating improved performance for automated medicinal plant identification. Do‐it‐yourself smart phone apps are also there for quickly identifying the plants like Flora Incognita, LeafSnap, etc. Yet in another development, a custom CNN was developed for plant leaf classification. The architecture includes six convolutional layers with associated max‐pooling and dense layers. The model was evaluated on three datasets: the Indian Medicinal Leaves Image Dataset, the MED117 Medicinal Plant Leaf Dataset, and a self‐curated dataset. Using optimizers such as Adam, RMSprop, and SGD with momentum, the model achieved high classification performance with accuracies of 99.5%, 98.4%, and 99.7%, respectively, demonstrating its effectiveness for medicinal plant identification (Chetia et al. 2025).

Beyond the physical authentication of botanical materials via image analysis, AI is also fundamentally restructuring the upstream ethnobotanical mining process by shifting the initial selection of candidate plants from anecdotal curation to evidence‐based prediction. This virtual screening utilizes NLP and knowledge graphs to mine vast repositories of ethnobotanical texts and scientific literature, linking traditional uses to modern molecular targets. For instance, Yoo et al. (2020) successfully employed a DL framework to analyze over 4500 plant‐derived natural compounds. Overall, AI‐based plant identification has achieved impressive classification accuracy and has significantly improved the accessibility of taxonomic expertise. However, many reported models are trained and validated using curated image datasets collected under controlled conditions, which may not fully reflect field‐level variability. Performance can be affected by environmental factors, image quality, developmental stage, and interspecies similarity. Future studies should prioritize diverse real‐world datasets and independent validation to improve model robustness and practical applicability.

### 3.3 Extraction of Bioactive Compounds

Once the appropriate medicinal plant is accurately identified and selected utilizing AI‐guided visual and ethnobotanical tools, the immediate next challenge is efficiently optimizing the recovery of its bioactive constituents from the complex plant matrix. To reduce reliance on empirical trial‐and‐error, computational approaches, including ML, are increasingly being applied to optimize phytoconstituent extraction. By modeling the complex interactions between variables like solvent polarity, temperature, pressure, time, solid–liquid ratio, and particle size, these tools help guide and refine experimental design. ML models (e.g., ANNs, RF, and SVM) are used to predict extraction yield and selectivity. They are applied across techniques such as ultrasound, microwave, and pressurized liquid, and supercritical fluid extraction. These models also support rational solvent and solvent‐mixture selection. This includes the selection of greener alternatives. The predictions are based on physicochemical descriptors (Liu et al. 2021).

Although predictive accuracies are high under controlled datasets, external validation across different laboratories and plant batches remains necessary. When coupled with spectroscopic process and chromatographic data, DL enables real‐time monitoring of extraction progress, early detection of degradation, and dynamic termination at optimal recovery, improving reproducibility and resource efficiency (Hu and Qiu 2023). In parallel, data‐driven models assist in anticipating scale‐up effects and batch‐to‐batch variability, while NLP‐based mining of phytochemical and ethnobotanical literature helps prioritize extraction strategies for specific metabolite classes, collectively streamlining phytoconstituent extraction without replacing underlying extraction chemistry.

Translating these broad predictive capabilities into specific laboratory applications, researchers are actively deploying ML to refine distinct, high‐energy extraction techniques. To overcome inefficient conventional extraction methods, ML was applied to optimize microwave‐assisted extraction (MAE) of phenolics and tannins from *Punica granatum* peel. Utilizing data from 30 varied MAE experiments, an LSBoost model combined with RF was deployed to evaluate predictive performance. This methodology achieved exceptional accuracy (*R* ^2^ = 0.9998) and identified microwave power as the primary optimization parameter, effectively overcoming the limitations of traditional low‐yield techniques to meet the demand for precision‐optimized bioactive compound recovery (Mobasheri et al. 2025).

Beyond microwave‐driven processes, ML models are proving equally highly effective in optimizing acoustic cavitation techniques. For instance, to enhance the extraction of bioactive phytocompounds from *Solanum torvum* using ultrasound‐assisted solvent extraction (UASE), ML‐assisted optimization was applied. The methodology combined a response surface methodology with an adaptive neuro‐fuzzy inference system and RF models to optimize five parameters: methanol concentration, ultrasound intensity, temperature, time, and particle size. This approach effectively addresses the gap in traditional extraction efficiency, with optimal conditions (65% methanol, 60 W cm^−2^, 45°C, 15 min, 0.5 mm) yielding high total polyphenols (830.68 mg GAE/g) and flavonoids (535.38 mg RE/g). Model predictions closely matched experimental results, and GC–MS/LC–MS analyses confirmed diverse bioactive constituents in the optimized extract (Petchimuthu et al. 2023).

While these single‐technique models demonstrate high predictive accuracy, AI frameworks are also being scaled to replace rigid traditional statistical methodologies across highly complex, multi‐stage extraction pipelines. To overcome the limitations of conventional orthogonal experimental design (OED) in complex extraction processes, an ML‐assisted framework was developed. Integrating quantitative ^1^H NMR, HPLC fingerprints, molecular weight, yield, and component data, the methodology employed grey relational analysis, RBF neural networks, a genetic algorithm, and CRITIC–TOPSIS ranking to optimize water and ethanol conditions. Compared with conventional OED, the ML‐assisted strategy achieved improved optimization performance, with low experimental deviation (1.33%–30.11%) and successful characterization of 160 compounds by UHPLC–MS/MS (Ma et al. 2023).

### 3.4 Identification of Phytoconstituents and Dereplication

Following the extraction of botanical material, the identification and structural elucidation of bioactive compounds represent a crucial yet traditionally challenging bottleneck in MPDD. While analytical techniques such as nuclear magnetic resonance (NMR) and mass spectrometry (MS) are standard for determining molecular structures, the manual interpretation of these complex spectra is highly specialized, time‐consuming, and susceptible to human error (Zani and Carroll 2017). To overcome this, scientists are increasingly deploying ML models trained on expansive chemical databases to automate spectral analysis. These computational models can rapidly match known compounds and even predict novel molecular structures directly from MS data, significantly reducing manual effort. Furthermore, AI excels at integrating multimodal analytical data, merging results from HPLC, GC–MS, and IR spectroscopy to generate a comprehensive phytochemical profile. This multi‐instrument integration ensures accurate characterization and minimizes the risk of overlooking potentially active, low‐abundance constituents.

Within this advanced analytical framework, molecular networking serves as a highly effective, complementary tool for processing complex tandem mass spectrometry (MS/MS) data (Brittin et al. 2025). Molecular networking computationally organizes this mass spectral data into relational clusters, enabling the easy visualization of structural similarities across a plant's entire metabolome (Nothias et al. 2018). A primary advantage of molecular networking is its capacity for rapid dereplication; by instantly comparing experimental spectra against public databases like GNPS, it systematically filters out previously characterized phytochemicals. Consequently, the common trap of rediscovering known metabolites can be bypassed, redirecting focus toward isolating uncharacterized chemical dark matter.

Crucially, the combination of multi‐omics data integration and molecular networking creates the structured data layer necessary to feed downstream AI predictive models and network pharmacology platforms. Once novel plant‐based scaffolds are elucidated, AI can forecast their biological activity by comparing the new chemical structures against databases of known bioactives. By coupling this clustered spectral data with ML algorithms and virtual screening techniques, rapid *in silico* molecular docking can be executed to match plant‐derived compounds with specific disease‐related protein targets. This integrated pipeline not only highlights which compounds should be prioritized for physical testing but also drastically reduces the time and financial investment required to translate crude botanical extracts into viable, targeted therapeutic leads (Hu and Qiu 2023; Wolfender et al. 2019).

Putting these integrated computational pipelines into practice, recent ML frameworks have specifically targeted the structural elucidation bottleneck inherent in mass spectrometry. To address the prevalent challenge of compound rediscovery in natural product drug discovery, an ML framework integrating LC–MS/MS‐based untargeted metabolomics and in silico fragmentation was developed. Utilizing molecular fingerprints generated by SIRIUS 5, the methodology predicted compound drug classes directly from MS/MS spectra without requiring reference experimental spectra. The framework demonstrated classification performance across 21 bioactive drug classes (> 93% accuracy), facilitating the rapid identification of promising antibacterial and antifungal natural products (Brittin et al. 2025).

While MS provides rapid untargeted profiling, the resolution of intricate stereochemistry necessitates similarly advanced DL interventions for NMR data. To improve the robustness of 2D NMR‐based dereplication and structure elucidation against spectral artefacts and solvent effects, a CNN‐based tool named SMART was developed. Integrating non‐uniform sampling (NUS) 2D NMR with DL, the model was trained on 2054 HSQC spectra utilizing contrastive loss. This methodology employed an optimized NUS‐HSQC protocol to effectively cluster newly isolated compounds with known analogues in an embedded spectral space (Zhang et al. 2017).

Moving beyond mere structural identification, researchers are now leveraging these robust chemical profiles to directly forecast biological activity. For instance, to bypass the need for prior in vitro biological testing, an ANN was developed to predict the anti‐inflammatory (COX‐1/5‐LOX) potential of asteraceae plant extracts based solely on their LC–MS chemical profiles. Utilizing genetic algorithms and decision trees, the methodology isolated 11 key biomarkers from over 6000 chromatographic peaks to train the model. With 81% accuracy and a perfect precision rate of 100%, the resulting ANN overcomes the limitations of traditional screening by identifying bioactive potential straight from complex chemical data (Chagas‐Paula et al. 2015).

Although AI‐assisted dereplication has substantially improved the speed of phytochemical annotation, most reported studies rely on reference databases that remain incomplete for many medicinal plants. Consequently, the performance of these models may decline when analyzing rare or previously uncharacterized metabolites. Furthermore, differences in spectral acquisition protocols and database coverage can affect reproducibility across laboratories. Future efforts should focus on standardized spectral repositories and broader representation of plant metabolomes to improve model robustness and generalizability.

### 3.5 ADMET Prediction and Virtual Screening

After isolating and characterizing novel plant‐based scaffolds through AI‐driven molecular networking, computational triage via virtual screening and ADMET prediction becomes essential to prioritize candidates with the safest and most favorable pharmacokinetic profiles prior to physical testing. AI is significantly advancing ADMET prediction in MPDD by addressing the intrinsic complexity of phytochemicals, which are structurally diverse, polyfunctional, and often underrepresented in conventional synthetic‐drug datasets (He et al. 2019). AI‐driven virtual screening frameworks are increasingly employed at the earliest stages to rapidly filter large libraries of plant‐derived compounds based on predicted target affinity, drug‐likeness, and preliminary ADMET suitability (Xu et al. 2025).

Within these pipelines, ML models such as RF, SVM, and gradient boosting algorithms learn nonlinear relationships between molecular descriptors and key ADMET endpoints. Molecular descriptors include physicochemical properties, topological indices, and molecular fingerprints. Key ADMET endpoints include intestinal absorption, blood–brain barrier permeability, CYP450 inhibition, and hepatotoxicity. This enables high‐throughput in silico prioritization of phytochemicals. Such prioritization can be performed before resource‐intensive wet‐lab experiments.

DL architectures, including GNNs, CNNs, and transformer‐based models, further enhance virtual screening and ADMET prediction by directly learning from molecular graphs or SMILES representations, allowing the capture of subtle structural motifs characteristic of various plant metabolites (Bai et al. 2025; Cobre et al. 2023). Transfer learning and multitask learning strategies, though specific examples are rare, are particularly valuable for medicinal plant research, as models pre‐trained on large synthetic‐compound datasets can be fine‐tuned using smaller phytochemical datasets to improve predictive robustness under data‐scarce conditions (Gangwal, Ansari, Ahmad, Azad, and Sulaiman 2024). Additionally, AI models are being integrated with metabolic pathway and toxicity prediction tools to forecast biotransformation routes, identify potentially harmful metabolites, and flag herb–drug interaction risks during virtual screening itself (Spanakis et al. 2025). Combining AI‐based virtual screening with ADMET prediction provides an early‐stage triaging step in MPDD. This approach aims to reduce attrition by prioritizing phytoconstituents with drug‐like properties, supporting the progression from complex plant extracts to natural product leads. Here, we summarize reported applications of AI in ADMET prediction and virtual screening.

ML‐based virtual screening using multilayer perceptron and RF models identified anti‐obesity candidates from medicinal plants targeting pancreatic lipase and AMPK. Both models showed strong predictive performance. Gypenoside LXVI and alisol‐B‐23‐acetate emerged as dual‐target hits and were experimentally validated, demonstrating lipase inhibition (IC_50_ 359.7 and 433.8 μg/mL) and significant reduction in lipid accumulation. Docking confirmed stable target interactions, highlighting the effectiveness of ML‐driven screening for plant‐derived anti‐obesity compounds (Zhou et al. 2024).

Samad et al. (2023) reported an integrated computational strategy in which ML‐driven virtual screening was coupled with molecular dynamics (MD) simulations to assess the antiviral potential of natural products against SARS‐CoV‐2. In their study, a RF model was trained on molecular descriptor data derived from thousands of phytochemicals compiled from existing literature. This approach enabled the prioritization of candidate compounds with a high probability of bioactivity, particularly those capable of targeting and inhibiting a key viral protease essential for SARS‐CoV‐2 replication. To identify promising therapeutic leads for cancer research, 11,908 natural compounds from the IMPPAT‐2 database were screened against the ABL2 tyrosine kinase. Utilizing structure‐based virtual screening, physicochemical and ADMET filtering, the methodology identified Pachyrrhizin and Lupinisoflavone K as top candidates (Elayyan et al. 2025).

To identify therapeutic candidates against SARS‐CoV‐2, 271 phytochemicals from Himalayan plants were screened against nine viral proteins. By combining a hybrid ML model (k‐nearest neighbor classifier) with molecular docking and dynamics simulations, the computational pipeline evaluated binding affinities and pharmacokinetic properties. This integrated approach successfully addresses the gap in rapid screening workflows, identifying Cepharadione A from *Piper longum* as a highly stable, multi‐target lead candidate with strong binding and favorable pharmacokinetic profiles (Maiti et al. 2024).

Several additional studies have further demonstrated the utility of AI‐driven virtual screening and ADMET prediction in identifying plant‐derived lead compounds for diverse therapeutic applications, including hyperuricemia, Alzheimer's disease, hepatocellular carcinoma, oral squamous cell carcinoma, cancer, and cholera (An et al. 2025; Islam et al. 2024; Jain et al. 2025; Randhawa et al. 2015; Jia et al. 2022; Bajrai et al. 2025).

In summary, AI‐assisted virtual screening and ADMET prediction provide an effective computational prioritization framework for MPDD by rapidly prioritizing phytoconstituents with favorable drug‐like and safety profiles from large and chemically diverse natural‐product libraries. These approaches significantly enhance screening efficiency and reduce the number of candidates requiring experimental evaluation. However, most studies continue to rely heavily on computational endpoints, including docking scores, predicted pharmacokinetic properties, and molecular dynamics simulations, with limited experimental confirmation. In addition, variations in training datasets, molecular descriptors, and validation protocols hinder direct comparison of model performance, while the frequent use of synthetic‐drug datasets may limit applicability to structurally complex phytochemicals. Therefore, future research should emphasize standardized benchmarking, prospective validation, and the integration of experimentally generated phytochemical datasets. Most importantly, AI‐generated predictions must be routinely supported by robust in vitro and in vivo studies to confirm biological activity, evaluate safety, and enhance the translational reliability of AI‐driven natural product discovery.

### 3.6 Repurposing

While virtual screening accelerates the de novo discovery of new therapeutic leads from complex extracts, AI predictive modeling also offers a highly efficient, alternative pathway by screening previously established phytoconstituents for entirely new therapeutic indications. Drug repurposing, also known as drug repositioning, is the strategy of identifying new therapeutic uses for existing approved, discontinued, or investigational drugs outside of their originally intended medical indication (Gangwal and Lavecchia 2024). Because the safety, dosage, and toxicity profiles of these compounds have already been rigorously established in previous clinical trials, this approach bypasses the lengthy and costly early stages of traditional development. Consequently, repurposing significantly reduces the time, financial risk, and failure rates associated with discovering new treatments, offering a much faster route to clinical use for various diseases (Chaachouay and Zidane 2024).

Applying the drug repurposing approach to established herbal molecules significantly accelerates the development timeline. Beyond saving time and resources, this strategy capitalizes on the natural, multi‐target efficacy of these unique plant‐derived structural scaffolds, making them exceptionally suited for treating complex, systemic conditions and overcoming established drug‐resistance mechanisms. Whether utilized as standalone treatments or in synergistic combinations with conventional synthetic therapies, repositioning these proven phytoconstituents provides a highly efficient and cost‐effective pathway to unlocking novel therapeutics (Rudrapal et al. 2022). Repurposing leverages established safety profiles, multitarget pharmacology, and polyvalent interactions of these natural scaffolds, exemplified by curcumin's pivot from anti‐inflammatory (Peng et al. 2021) to antiviral agent against SARS‐CoV‐2 (Zahedipour et al. 2020) or quercetin's repurposing for neuroprotective effects (Adnan et al. 2025). AI significantly enhances this paradigm by harnessing vast phytochemical databases for predictive modeling. ML‐driven QSAR analyses and network pharmacology dissect polypharmacology, identifying off‐target efficacies; for instance, RF and SVM prioritize candidates like apigenin for glioblastoma via virtual screening.

To identify COVID‐19 therapeutics via a repurposing strategy, 2431 phytochemicals from 104 Korean medicinal plants were screened against the SARS‐CoV‐2 main protease (Mpro). The methodology integrated docking followed by recurrent neural networks (RNN)‐based re‐screening to isolate 12 high‐affinity candidates (−8.0 to −8.9 kcal/mol). Catechin gallate and quercetin 3‐O‐malonylglucoside emerged as lead candidates, demonstrating stable target interactions in MD simulations and favorable ADMET profiles suggestive of repurposing potential (Hossain et al. 2023). Another repurposing study explored phytochemicals as dual‐target antifungal agents against sterol‐14‐α‐demethylase and HSP‐90. Screening 1149 ligands from 45 medicinal plants identified 83 dual‐active hits, with isocarthamidin, quercetin, and boeravinone B selected based on docking performance relative to fluconazole and ganetespib. Subsequent MM/GBSA and MD analyses confirmed stable target interactions, supporting their potential as dual‐target antifungal candidates (Kaur et al. 2024).

To accelerate drug repositioning, an RF ML model predicted new therapeutic indications for 20 existing drugs and 31 herbal compounds. By integrating drug and disease similarity features, the methodology identified potential uses that were subsequently validated using clinical trial data. By integrating drug and disease similarity features, this computational strategy facilitated the identification of potential therapeutic applications for under‐studied natural products (Kim et al. 2019). Nonetheless, repurposed phytochemicals must still undergo rigorous pharmacokinetic, toxicological, and clinical evaluation, limiting the extent to which development timelines can be shortened.

### 3.7 De Novo Drug Design

Beyond finding new clinical applications for existing plant molecules, GAI is pushing the boundaries of phytomedicine by utilizing these complex natural scaffolds as foundational templates to computationally build entirely novel, highly synthesizable pseudo‐natural products from scratch. De novo drug design is a sophisticated computational approach that builds entirely novel chemical entities from scratch, atom by atom or fragment by fragment, based on the three‐dimensional structure of a specific biological target or the properties of known active ligands, rather than relying on the traditional screening of pre‐existing compound libraries (Janet et al. 2023). The primary benefit of this strategy in drug discovery is its ability to explore a virtually infinite chemical space, enabling the creation of highly optimized molecules with precise binding affinities, superior pharmacokinetic profiles, and minimized off‐target toxicity (Gangwal, Ansari, Ahmad, Azad, Kumarasamy, et al. 2024).

In MPDD, researchers are leveraging this approach by using the complex, privileged structures of established phytoconstituents as foundational templates rather than just final products. By deploying advanced GAI, scientists can computationally evolve these natural scaffolds to generate vast libraries of optimized semi‐synthetic analogues, fully synthetic derivatives, and innovative pseudo natural products (Zhang, Yang, et al. 2025). These AI‐generated pseudo natural products are particularly valuable. They recombine natural product fragments in non‐natural arrangements. This approach bridges the evolutionary advantages and structural complexity of medicinal plants with the drug‐likeness and synthesizability required for modern pharmaceuticals. It can help overcome traditional limitations such as poor bioavailability. At the same time, it maintains therapeutic efficacy (Bhadra et al. 2025). While traditional AI (discriminative models) focuses on recognizing plants or predicting if a known compound is active, GAI models [such as transformers, GANs, VAEs, and diffusion models] can create entirely new molecular structures (which may or may not have promising features).

By translating these discriminative recognitions into generative actions, advanced computational frameworks directly address the most prohibitive barriers of botanical research: structural intractability and limited physical supply. By treating chemical structures like language (using strings of text) or graphs, GenAI can read the complex scaffolds of plant metabolites and write new, simplified synthetic analogues (pseudo‐natural products) that retain the biological potency of the plant but are easier to synthesize and safer for human use. This accelerates scaffold exploration compared to traditional manual approaches. Here we present a few successful examples of GAI applications in MPDD.

Demonstrating the power of this language‐based molecular generation, recent transformer‐based architectures have been successfully engineered to intentionally fragment and recombine complex phytoconstituents. To improve synthetic accessibility while retaining biological relevance, a natural product‐inspired molecular generative model (NIMO) based on a conditional transformer was developed. By decomposing complex natural products into functional fragments, the model generates pseudo‐natural molecules. This approach effectively advances structure‐based pseudo‐natural product design, outperforming conventional baselines with a 95.4% success rate in generating complex terpenoids, achieving 55.9% predicted antimalarial activity, and producing pocket‐specific antibacterial candidates with superior docking scores (Shen et al. 2024).

Beyond transformer‐based fragmentation, alternative generative methodologies prioritize absolute synthetic feasibility by computationally assembling natural scaffolds through established, reaction‐driven algorithms. To ensure synthetic accessibility in de novo drug design, a ligand‐based tool named DOGS (Design of Genuine Structures) was developed. Utilizing a reaction‐driven assembly of approximately 25,000 building blocks and 58 established reactions, the methodology evaluates generated candidates with defined synthetic routes via graph kernel similarity to reference ligands. This study provides a robust framework for incorporating practical synthetic feasibility into molecular design, confirming its scaffold‐hopping and drug‐like design capability by successfully generating bioactive compounds targeting γ‐secretase and the human histamine H₄ receptor (Hartenfeller et al. 2012).

Complementing these reaction‐driven assemblies, RNNs offer an independent generative pathway by mathematically embedding native plant characteristics directly into synthetic training spaces. RNNs enable the de novo design of synthesizable chemical entities by embedding natural product characteristics into models trained on synthetic compounds. This generative approach was validated through the identification of computer‐generated retinoid X receptor (RXR) modulators, which demonstrated isofunctional activity in vitro. Such models effectively expand the accessible chemical space for natural‐product‐inspired drug discovery, offering a streamlined pathway for identifying novel, synthesizable therapeutic scaffolds (Merk et al. 2018).

To further expand this accessible chemical space while actively optimizing for pharmacokinetic viability, researchers are combining advanced generative pre‐trained transformers with active optimization search strategies. To address the restricted chemical space and poor drug‐likeness of traditional AI‐driven models, the NPDL‐GEN framework, integrating GPT1 with Augmented Hill‐Climb (AHC) strategies, was developed to generate diverse, synthetically accessible pseudo‐natural products. This model successfully identified leads G1–G5 with optimized pharmacokinetic profiles and anti‐inflammatory candidates H1–H3 via transfer learning. By maintaining the structural integrity of natural templates while enhancing molecular novelty and validity, the NPDL‐GEN platform provides a high‐efficiency pathway for accelerating natural product‐inspired drug discovery (Lu et al. 2025).

Utilizing the AI‐driven TransGenGRU model, Lin et al. (2025) generated novel natural product derivatives targeting the NLRP3 inflammasome, identifying guaianolide sesquiterpenoids as promising scaffolds. The optimized lead demonstrated a unique covalent‐irreversible interaction with Cys280, a binding mechanism distinct from the conventional inhibitor MCC950. Supported by favorable DMPK profiles and low hERG toxicity, E1 exhibited potent in vivo anti‐inflammatory activity, establishing it as a highly effective and novel candidate for therapeutic intervention in inflammasome‐mediated diseases. Despite promising in silico performance, many generated pseudo‐natural products still face challenges in synthesis feasibility, regulatory approval, and in vivo validation.

Despite these targeted in silico successes, the broader translational deployment of GAI for medicinal plant remains severely overshadowed by repurposing efforts and constrained by persistent algorithmic bottlenecks. While recent corona crises spurred a surge of AI deployment in drug repurposing, the generative design of novel phytochemicals remains critically underexplored. Deploying GAI for MPDD is currently bottlenecked by the structural complexity of plant metabolites and algorithmic flaws like mode collapse and chemical hallucinations, which yield repetitive or biologically impossible structures. Despite this scarcity, customizing GAI for botanical discovery offers immense untapped potential. By shifting to knowledge‐guided frameworks, researchers can design highly synthesizable, pseudo‐natural analogs that retain the evolutionary binding advantages of plant scaffolds while overcoming the supply and structural limitations of traditional botanical drug development.

### 3.8 Network Pharmacology/Target Identification

Whether investigating native plant compounds, repurposed molecules, or AI‐generated pseudo‐natural derivatives, understanding how these multi‐component entities synergistically modulate complex biological systems requires the holistic, AI‐mapped approach of network pharmacology. Network pharmacology is an interdisciplinary approach that combines systems biology and bioinformatics to map the complex, web‐like interactions between drugs, biological targets, and diseases, moving away from the traditional one drug, one target model (Hopkins 2008).

**FIGURE 5:**
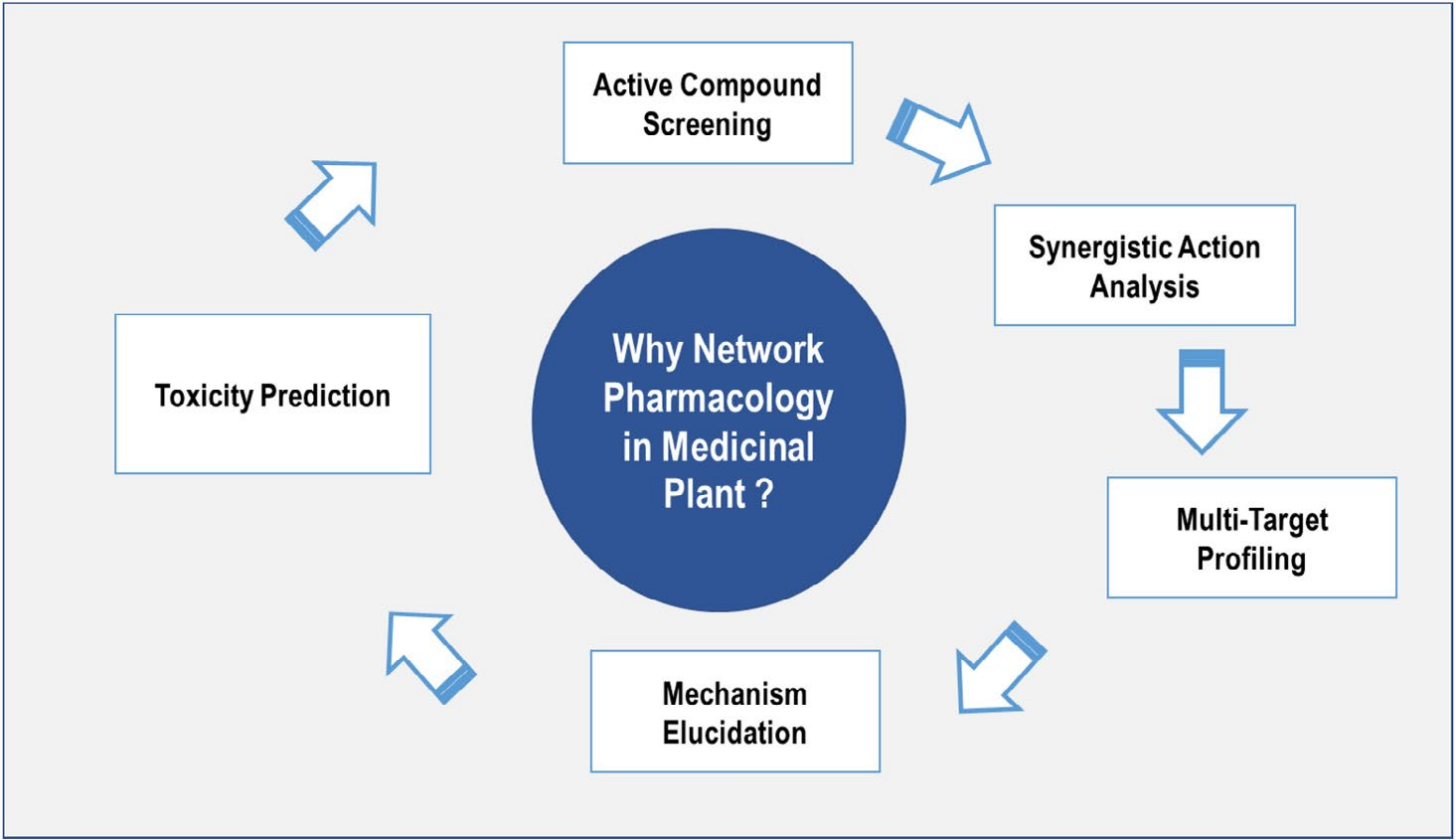
Applications of network pharmacology in MPDD.

This holistic perspective makes it exceptionally important for phytomedicine, as medicinal plants naturally contain dozens or hundreds of bioactive compounds that exert their therapeutic effects through synergistic interactions across multiple biological pathways rather than hitting a single receptor (Figure 5). By mapping complex systems like protein–protein interactions and metabolic pathways, these models can predict both promising plant compounds and their likely therapeutic targets for treating complex diseases (Gangwal et al. 2026).

AI has substantially expanded the analytical capabilities of network pharmacology. It has facilitated a shift from the reductionist one‐drug, one‐target model toward a holistic multi‐component, multi‐target approach. This approach is tailored to the inherent complexity of medicinal plants. By leveraging advanced computational architectures such as GNNs, AI systematically maps the intricate herb‐compound‐target‐disease (H‐C‐T‐D) interactome, integrating vast datasets from phytochemical databases and protein–protein interaction networks. These models are uniquely capable of decoding pharmacological synergy, often termed the cocktail effect, by predicting how diverse plant constituents concurrently modulate multiple biological nodes within a disease pathway.

Beyond target identification, AI‐guided network analysis simulates the perturbation of metabolic and signaling networks to identify lead scaffolds that offer superior therapeutic efficacy and reduced toxicity compared to isolated compounds. Furthermore, the integration of AI‐driven ADMET profiling within these networks allows for the prediction of systemic herb‐drug interactions and the pharmacokinetic behavior of complex extracts. This AI‐mediated systems biology approach provides a structured computational framework that can support the validation of traditional herbal knowledge when combined with experimental evidence (Cui et al. 2025).

Acetylbinankadsurin A (ACBA) from *Kadsura coccinea* showed significant anti‐fibrotic effects in CCl_4_‐induced liver fibrosis models, reducing collagen deposition, α‐SMA expression, and inflammatory markers. Network pharmacology and ML analyses highlighted NF‐κB signaling and identified CCR1 as a key target. Docking, MD, and experimental validation confirmed ACBA–CCR1 interactions and suppression of NF‐κB/STAT1 activation, supporting its potential as a natural anti‐fibrotic agent (Peng et al. 2024).

Several other studies have similarly demonstrated the utility of AI‐integrated network pharmacology, often in combination with ML, DL, molecular docking, molecular dynamics simulations, AlphaFold, and multi‐omics approaches, for target identification, mechanism elucidation, and candidate prioritization in medicinal plant‐based drug discovery (Liu et al. 2024; Fu et al. 2023; Chao et al. 2022; Zhang, Zheng, et al. 2025; Galkin et al. 2025).

The trajectory of these integrations highlights a radical shift in the field; indeed, a recent survey describes how prompt to drug (via LLM) has the potential to revolutionize the entire drug discovery pipeline (Zhavoronkov et al. 2026). Nevertheless, these emerging GAI and prompt to drug frameworks should be interpreted with appropriate caution. Despite impressive advances in LLMs, foundation models, and autonomous AI agents, most reported applications remain confined to computational proof‐of‐concept studies, with limited experimental or clinical validation. The ability of these systems to reliably generate biologically relevant, synthetically accessible, and pharmacologically viable candidates remains an active area of investigation.

Moving forward, the systematic deployment of advanced structural biology tools like AlphaFold3 will be critical for decoding the precise targets and modes of action of complex phytoconstituents. By accurately predicting the 3D conformations of previously uncharacterized or orphan receptor proteins, one can move beyond the limitations of traditional molecular docking, which relies exclusively on known crystallographic structures, and deeply investigate how novel plant‐derived scaffolds interact with complex disease networks at an atomic level.

A comparison of recent studies suggests that network pharmacology currently represents a more mature and experimentally supported application of AI in phytomedicine than generative de novo design. Network‐based approaches benefit from established biological interaction databases and provide mechanistic insights into the multi‐target nature of medicinal plants, whereas GAI remains largely confined to in silico proof‐of‐concept studies. Although transformer models, GANs, and VAEs have demonstrated the ability to generate structurally novel pseudo‐natural products, challenges related to synthetic feasibility, biological relevance, and experimental validation remain substantial. Consequently, the greatest near‐term impact of AI in medicinal plant research is likely to arise from the integration of network pharmacology, multi‐omics data, structural biology, and predictive modeling rather than from fully autonomous molecular generation alone.

## 4 Limitations, Challenges, and Translational Barriers of AI in MPDD

The successful implementation of AI in MPDD is significantly hindered by both inherent algorithmic limitations and domain‐specific botanical complexities. Predictive models frequently struggle with poor data digitization, taxonomic ambiguities, batch‐to‐batch variability, and the intricate multi‐component nature of herbal formulations. To transition from exploratory computational exercises to robust translational tools, researchers must first systematically resolve these foundational data constraints. Table 3 systematically categorizes these core architectural and methodological bottlenecks, proposing actionable pre‐processing frameworks to render complex phytochemical inputs truly AI‐ready and detailing the enhanced predictive capabilities achieved through such infrastructural reforms.

**TABLE 3:** Architectural and methodological challenges in AI‐enabled MPDD: causes, corrective frameworks, and predictive outcomes.

Challenge	Reason for challenge	First step: making input AI‐ready	How AI addresses it (once data is ready)
Data redundancy & algorithmic bias	Over‐representation of celebrity plants (e.g., *Curcuma*) and common secondary metabolites (e.g., plant sterols, quercetin, kaempferol, etc.) causes overfitting and ignores rare/relatively less explored species	Pre‐filter datasets to down‐sample overrepresented species/compounds and up‐sample rare ones. Explicitly tag and weight common metabolites to lower their predictive influence	Trained on balanced data, the AI avoids popularity bias, accurately predicting bioactivity for understudied species rather than just regurgitating known data
Conflicting annotations & noise	Lack of standardized protocols leads to divergent bioactivity reports for the same species due to varied solvents or assays	Implement a strict metadata framework (e.g., standardizing assay types, solvent codes) to label experimental conditions distinctively before feeding the model	AI distinguishes between true biological variance and experimental artifacts, identifying stable pharmacological signals amidst noise
Obstruction of novelty detection	Saturation of training sets with known plant‐lead associations conditions models to prioritize known scaffolds over new ones	Create Novelty‐First datasets by masking well‐known associations and explicitly flagging dark matter (unstudied) data points during pre‐processing	The model shifts focus from pattern matching to anomaly detection, flagging unique chemical structures that do not fit established profiles as potential new drugs
Organ & extraction variability	Metabolites vary by plant part (root vs. leaf) and extraction solvent, causing apparent inconsistencies in data	Tag every data point with specific Part Used and Extraction Method fields. Treat different parts/extracts as distinct inputs, not just generic Species X	AI learns to predict bioactivity specific to the plant part (e.g., Root Extract vs. Leaf Extract) rather than a generalized (and inaccurate) species‐level prediction
Taxonomic ambiguity	Use of synonyms, vernacular names, or misidentified species creates duplicate or erroneous entries	Clean datasets using a standardized botanical reference to resolve synonyms and correct misidentifications before ingestion	The AI treats synonymous names as a single entity, aggregating scattered data into a robust, single‐source‐of‐truth profile for each species
Publication positivity bias	Negative results are rarely published, leading AI to overestimate efficacy and underestimate toxicity	Actively mine and incorporate grey literature, theses, or inconclusive reports. Synthetic generation of plausible inactive samples can also balance the set	The model learns to penalize false positives and provides confidence intervals that reflect the probability of failure, not just success
Incomplete metabolomic data	Studies report only abundant compounds, creating an illusion that the list is complete	Tag datasets with a Chemical Coverage Score. Use untargeted metabolomics data where available to represent the full spectrum	AI infers the presence of hidden minor constituents based on the presence of major ones, predicting bioactivity driven by the whole plant rather than just reported peaks

While improving data quality and reducing algorithmic bias are essential prerequisites for reliable AI implementation, an equally important challenge lies in translating computational predictions into experimentally validated therapeutic outcomes. Despite impressive advances in virtual screening, ADMET prediction, network pharmacology, and GAI, many compounds identified as promising candidates during in silico analyses ultimately fail during subsequent biological evaluation. This translational gap remains one of the most significant obstacles preventing the routine integration of AI into MPDD.

Several factors contribute to this discrepancy. First, computational approaches such as molecular docking and ML‐based activity prediction frequently rely on simplified representations of biological systems and may not adequately capture protein flexibility, allosteric regulation, cellular context, or complex disease biology. Consequently, high docking scores or favorable prediction probabilities do not necessarily translate into measurable biological activity. Second, many AI models are trained predominantly on synthetic‐drug datasets and may therefore exhibit reduced predictive accuracy when applied to structurally diverse phytochemicals possessing unique stereochemistry, scaffold complexity, and multi‐target pharmacology.

The challenges become even more pronounced during in vitro and in vivo validation. Phytochemicals predicted to be active may exhibit poor aqueous solubility, limited membrane permeability, rapid metabolic degradation, inadequate bioavailability, or unanticipated toxicity that was not evident during computational screening. Furthermore, medicinal plants often exert therapeutic effects through synergistic interactions among multiple constituents, whereas most AI frameworks evaluate compounds individually. As a result, isolated phytochemicals may fail to reproduce the biological activity observed in complex extracts or traditional formulations. These limitations highlight the necessity of integrating AI predictions with rigorous phytochemical characterization, standardized biological assays, pharmacokinetic evaluation, and experimental validation to establish genuine translational relevance.

Future progress will depend not only on algorithmic advances but also on the development of standardized phytochemical databases, transparent reporting practices, independent external validation studies, and experimentally grounded benchmarking frameworks. Such efforts will improve reproducibility, reduce model bias, and facilitate the successful translation of AI‐generated hypotheses into clinically meaningful medicinal plant‐derived therapeutics.

## 5 Future Directions

As the field looks to the future, research must intentionally pivot away from saturated sub‐domains. Driven by the urgency of global health crises and the availability of accessible datasets, a disproportionate volume of recent literature has focused heavily on AI models for medicinal plant identification, virtual screening, ADMET prediction, and COVID‐19 drug repurposing.

While these studies served as vital stepping stones, the community must now direct AI applications toward resolving the more complex, fundamental facets of botanical drug discovery, such as extraction optimization, decoding multi‐component synergy, and illuminating the uncharted chemical dark matter of plant metabolomes. Building on the limitations identified in this timely review, we suggest that the next phase of AI‐driven MPDD research must begin with making phytochemical data truly AI‐ready. Algorithmic sophistication alone will not correct deeply rooted issues such as data redundancy, annotation noise, taxonomic ambiguity, or publication bias. Therefore, we propose that future efforts prioritize structured dataset engineering before large‐scale model deployment.

First, the development of phyto‐centric foundational models should be accompanied by rigorous dataset stratification and balancing strategies. The transformative success of AI in non‐pharmaceutical domains has been fundamentally driven by the deployment of robust, large‐scale foundation models (Table 4). To replicate this paradigm shift in phytomedicine, the medicinal plant drug discovery pipeline urgently requires the development of its own specialized foundation models, purpose‐built to navigate the unique structural complexities of natural products (Table 5).

**TABLE 4:** Landmark AI architectures, foundation models, and datasets.

Name	Broad category	Primary uses	What it changed in the AI world
AlexNet	Model architecture	Image classification	Proved the power of deep CNNs and GPUs, sparking the modern DL boom in 2012
AlphaFold	Foundation Model	3D protein structure prediction	Solved a 50‐year‐old grand challenge by predicting how proteins fold, drastically accelerating drug target identification
BBBC (Broad Bioimage Benchmark Collection)	Dataset	Cellular assay analysis	Standardized high‐quality fluorescent and cellular imagery. It allows researchers to test if an AI can accurately see how cells react to different chemical compounds
ChemBERTa/ChemGPT	Foundation Model	Molecular property prediction	Adapted natural language processing (Transformers) to read SMILES strings (chemical text), allowing AI to understand and predict the behavior of complex molecules
CheXpert/MIMIC‐CXR	Dataset	Medical diagnostics	Bridged the gap between computer vision and clinical health by providing hundreds of thousands of chest X‐rays paired with actual radiological reports
COCO (Common Objects in Context)	Dataset (Complex scenes)	Object detection & segmentation	Set the modern standard for evaluating how well AI understands complex, crowded, real‐world environments.
GPT/Gemini/Llama	Foundation Model	Text, reasoning, code	Popularized general‐purpose AI, proving one massively trained model could adapt to almost any language task
GNNs	Model Architecture	Molecular representation	Shifted AI from looking at 2D grids (like images) to analyzing the actual mathematical graphs of atoms and bonds in a molecule. Highly accurate for predicting toxicity and bioactivity
ImageNet	Dataset	Training vision models	Provided the massive, labeled scale required to make DL actually work, serving as the ultimate benchmark
MNIST	Dataset (Handwritten digits)	Education & basic ML testing	Became the universal Hello World dataset for computer science and AI debugging
MoLFormer	Foundation Model	Virtual screening & drug design	Enabled rapid, large‐scale screening of massive chemical libraries to identify viable candidates for pharmaceutical development
PlantVillage	Dataset (Plant leaves)	Agricultural AI/Botany	Democratized AI in agriculture by providing standardized data to train crop disease and species identification models
ResNet	Model Architecture	Deep image analysis	Introduced skip connections to solve the vanishing gradient problem, allowing networks to become hundreds of layers deep
U‐Net	Model Architecture	Biomedical image segmentation	Set the absolute standard for isolating precise biological structures (like individual cells or tumors in microscopy) using extremely small amounts of training data
ViT	Model Architecture	Advanced computer vision	Proved that transformer models (originally built for text) could outperform traditional CNNs on visual tasks
YOLO	Model Architecture	Real‐time object detection	Shifted object detection from a slow, multi‐step process to a single, lightning‐fast pass, enabling live video analysis

**TABLE 5:** Proposed AI foundation models for medicinal plant drug discovery.

Proposed foundation model	Model type	Primary function in medicinal plant drug discovery	Key limitation addressed
Model 1	Molecular Transformer	Learns representations of phytochemical structures to predict bioactivity and physicochemical properties	Existing AI models poorly capture natural product chemical space
Model 2	Multimodal Transformer + GNN	Connects plant species, metabolites, and biosynthetic pathways	Lack of integration between plant biology and metabolite data
Model 3	Structure‐based interaction model	Predicts binding between phytochemicals and disease targets	Traditional docking struggles with complex natural product structures
Model 4	Knowledge graph + LLM	Extracts drug leads from ethnobotanical and traditional medicine knowledge	Valuable traditional knowledge remains unstructured and underused
Model 5	GAI (Diffusion/GAN/Transformer)	Generates novel natural‐product‐like compounds and optimized analogs	Limited chemical diversity in existing natural product libraries

Current predictive systems are disproportionately trained on a small number of highly studied celebrity plants and recurrent metabolite classes, which reinforces algorithmic popularity bias and limits novelty detection. We recommend deliberate down‐sampling of overrepresented species and scaffolds, alongside up‐weighting of rare or underexplored taxa. Explicit tagging of common metabolite classes can reduce their predictive dominance.

When trained on balanced and stratified inputs, future phyto‐foundational models will be better positioned to identify biologically meaningful signals from understudied plants rather than merely reproducing known associations. Additionally, at present, most AI systems used in drug discovery are trained predominantly on synthetic compound libraries, which inevitably bias predictions toward chemically flat, nitrogen‐rich, and synthesis‐oriented scaffolds. Such models are not optimally aligned with the stereochemical richness, oxygenation patterns, glycosylation, and scaffold complexity typical of plant metabolites. We therefore suggest the development of multi‐modal foundational models trained specifically on medicinal plant‐derived compound datasets.

Second, we emphasize the urgent need for ontology‐level standardization to address conflicting annotations and experimental noise. Divergent assay conditions, inconsistent solvent reporting, and non‐standardized bioactivity metrics frequently distort training datasets. We suggest implementing strict metadata frameworks that encode assay type, extraction protocol, plant part, and concentration ranges as structured variables rather than free text. Treating different plant organs and extraction methods as distinct computational entities will enable context‐aware prediction rather than inaccurate species‐level generalization. Once harmonized, AI systems can begin to disentangle true pharmacological patterns from experimental artifacts.

Taxonomic harmonization must also become a foundational pre‐processing step. Synonyms, vernacular names, and misidentified species create duplicated or erroneous data entries that undermine model reliability. We recommend routine alignment of datasets with standardized botanical references prior to ingestion. Entity resolution strategies will allow AI systems to consolidate fragmented knowledge into unified species profiles, strengthening predictive robustness.

To overcome the obstruction of novelty detection, we propose the creation of novelty‐first training subsets. By masking well‐known plant–lead associations and explicitly flagging underexplored chemical space, models can be encouraged to shift from pattern memorization toward anomaly detection. This reframing will help AI identify structurally unique candidates that fall outside established phytochemical archetypes, thereby supporting genuine de novo hypothesis generation.

To achieve this unbiased baseline, there is an urgent need to develop robust, high‐throughput computational filters capable of rapidly identifying and penalizing ubiquitous, highly promiscuous phytochemicals, akin to pan‐assay interference compounds (PAINS) in synthetic screening. By filtering out these frequent hitters early in the data pre‐processing phase, investigators can prevent common, non‐specific metabolites from skewing model training, thereby ensuring the development of truly unbiased predictive models that accurately spotlight novel and specific bioactive scaffolds.

To overcome the critical bottlenecks of database fragmentation and literature saturation, the integration of advanced AI methodologies provides a robust, systematic solution. NLP algorithms can be deployed to mine decades of unstructured ethnobotanical literature, automatically extracting latent metabolomic data to reconcile conflicting chemical profiles across disparate repositories. Furthermore, structuring this harmonized data into comprehensive knowledge graphs allows researchers to mathematically map the intricate web of H‐C‐T‐D relationships. To practically navigate this vast data landscape, AI‐driven literature mapping platforms (like Research Rabbit and others) and similar visual discovery tools must be developed.

By dynamically generating interactive citation networks and conceptual mind maps, these applications allow researchers to intuitively visualize literature saturation and track the historical trajectory of specific botanical studies. This visual approach to literature review enables scientists to immediately pinpoint white spaces where a plant's pharmacological potential remains genuinely underexplored. Consequently, this combination of NLP data extraction and visual network mapping transforms the traditionally ambiguous task of plant selection into a precise, data‐driven strategy.

Publication positivity bias and incomplete metabolomic reporting represent additional structural distortions. We recommend active incorporation of negative results, grey literature, and inconclusive datasets into training corpora. Where empirical negative data remain scarce, carefully designed augmentation strategies may reduce false‐positive inflation. Similarly, tagging studies with a chemical coverage score can help models account for incomplete profiling. Advanced architectures may then infer latent minor constituents and estimate uncertainty bounds, producing more realistic efficacy predictions rather than binary optimism.

To operationalize these improvements, we envision the gradual emergence of semi‐autonomous, lab‐in‐the‐loop systems in which AI‐guided decision‐making is integrated with automated extraction, fractionation, and spectral analysis platforms. Such workflows would not only accelerate validation but also generate standardized, machine‐readable datasets necessary for continual model retraining. This iterative computational–experimental cycle is essential for moving beyond retrospective validation toward prospective discovery. The integration of agentic AI into medicinal plant research signifies a move toward the autonomous orchestration of the discovery pipeline, with inspiration drawn from recently published tools like ChatInvent by AstraZeneca (He et al. 2026). These systems utilize specialized agents to handle distinct tasks, offering a structured approach to address nearly every stage of the botanical discovery process through a multi‐agent architecture. Under such a framework, a supervisor agent could decompose complex research queries into specific sub‐tasks, delegating species authentication to a taxonomy‐focused agent, solvent optimization to an extraction agent, and spectral matching to a dedicated dereplication agent. This could extend to the generative design of pseudo‐natural products and the planning of their chemical assembly through retrosynthesis agents, all while being continuously monitored by scoring agents for ADMET profiling and bioactivity prediction.

However, it is important to maintain a balanced perspective. Synthetic drug discovery represents a relatively more predictable paradigm. Despite substantial advances in AI, no AI‐discovered drug has yet completed the full path to market approval. Medicinal plant research presents additional layers of complexity. Several of these challenges have been discussed throughout this paper. As of now, no fully AI‐driven clinically approved botanical drugs have emerged, and computational predictions remain strictly probabilistic. Therefore, agentic AI should be viewed not as a transformative guarantee of clinical success, but as a sophisticated supportive framework for enhancing decision‐making and data management in a field where rigorous experimental validation remains the ultimate and essential bottleneck.

Finally, to sustain this paradigm shift, the foundational training of researchers must evolve. Just as traditional pharmacognosy practicals, such as the microscopic study of transverse sections, biochemical estimation and isolation of secondary metabolites, are integral to pharmaceutical education, basic computational practicals driven by AI must now be incorporated into the curriculum. A pioneering initiative in this direction has been undertaken by the Pharmacy Council of India (PCI), a statutory body established under the Pharmacy Act of 1948 to regulate and promote pharmacy education, which has begun revamping the curriculum for the integration of these modern computational tools.

## 6 Conclusion

Medicinal plants represent an unparalleled, evolutionarily refined reservoir for drug discovery, yet their systematic translation into clinical therapeutics has historically been hindered by persistent bottlenecks discussed in this review. The integration of AI offers a pragmatic and necessary framework to overcome these intrinsic barriers. Across the discovery pipeline, AI interventions are actively reshaping traditional workflows.

Computer vision and NLP are resolving taxonomic ambiguities and mining ethnobotanical texts to move plant selection beyond serendipity. At the bench level, ML/DL are optimizing extraction variables and automating complex spectral interpretation to rapidly dereplicate known compounds, thereby preventing redundant isolation efforts. Furthermore, advanced systems modeling via GAI is enabling the de novo design of synthesizable pseudo‐natural products, while network pharmacology uniquely decodes the synergistic, multi‐target interactions of complex herbal formulations.

Despite these promising advancements, a pragmatic perspective remains essential: fully AI‐driven, clinically approved botanical or synthetic drugs have yet to emerge, and the field still faces several unresolved challenges. Current predictive models, predominantly trained on flat, nitrogen‐rich synthetic libraries, frequently struggle with the stereochemical richness, glycosylation, and macrocyclic scaffolds inherent to phytomedicine.

Furthermore, algorithmic biases often favor well‐studied plants, incomplete data digitization continues to obstruct true novelty detection, and the limited availability of standardized, experimentally validated phytochemical datasets restricts model reliability and translational applicability. Addressing these challenges will require practical measures, including the development of AI‐ready phytochemical repositories, harmonized metadata standards, balanced and novelty‐aware datasets, standardized benchmarking frameworks, and closer integration of computational predictions with experimental validation workflows.

Moving forward, the scientific community must pivot from isolated algorithmic exercises toward rigorous infrastructural data reform. Developing phyto‐centric foundational models, strictly harmonizing metadata, and curating novelty‐aware, balanced datasets are critical prerequisites for future success. Ultimately, AI serves as a sophisticated decision‐making facilitator rather than a replacement for traditional experimental workflows. Bridging advanced computational predictions with semi‐autonomous lab‐in‐the‐loop systems and rigorous wet‐lab validation will be the definitive key to transitioning from exploratory studies to the reproducible, next‐generation discovery of botanical therapeutics.

## Author Contributions

**Mohd Usman Mohd Siddique:** data curation, visualization. **Jyotiram Sawale:** investigation, formal analysis. **Raju Wadekar:** formal analysis. **Amit Gangwal:** conceptualization, writing – original draft, methodology, writing – review and editing, project administration, data curation, formal analysis, validation. **Azim Ansari:** visualization, data curation, resources. **Suhas Padmane:** formal analysis.

## Funding

The authors have nothing to report.

## Conflicts of Interest

The authors declare no conflicts of interest.

## Data Availability

Data sharing not applicable to this article as no datasets were generated or analysed during the current study.
